# In situ light‐activated materials for skin wound healing and repair: A narrative review

**DOI:** 10.1002/btm2.10637

**Published:** 2024-01-16

**Authors:** Jordan R. Yaron, Mallikarjun Gosangi, Shubham Pallod, Kaushal Rege

**Affiliations:** ^1^ Center for Biomaterials Innovation and Translation, The Biodesign Institute, Arizona State University Tempe Arizona USA; ^2^ School for Engineering of Matter, Transport, and Energy, Ira A. Fulton Schools of Engineering, Arizona State University Tempe Arizona USA; ^3^ Chemical Engineering, Arizona State University Tempe Arizona USA

**Keywords:** biomaterial, in situ, photoactivation, photoinitiation, wound dressing, wound healing

## Abstract

Dermal wounds are a major global health burden made worse by common comorbidities such as diabetes and infection. Appropriate wound closure relies on a highly coordinated series of cellular events, ultimately bridging tissue gaps and regenerating normal physiological structures. Wound dressings are an important component of wound care management, providing a barrier against external insults while preserving the active reparative processes underway within the wound bed. The development of wound dressings with biomaterial constituents has become an attractive design strategy due to the varied functions intrinsic in biological polymers, such as cell instructiveness, growth factor binding, antimicrobial properties, and tissue integration. Using photosensitive agents to generate crosslinked or photopolymerized dressings in situ provides an opportunity to develop dressings rapidly within the wound bed, facilitating robust adhesion to the wound bed for greater barrier protection and adaptation to irregular wound shapes. Despite the popularity of this fabrication approach, relatively few experimental wound dressings have undergone preclinical translation into animal models, limiting the overall integrity of assessing their potential as effective wound dressings. Here, we provide an up‐to‐date narrative review of reported photoinitiator‐ and wavelength‐guided design strategies for in situ light activation of biomaterial dressings that have been evaluated in preclinical wound healing models.

**Translational Impact Statement**

Dermal wounds are a major medical burden, and there is an unmet need for novel therapies to improve complex wound healing. Biomaterial wound dressings have become a popular design strategy for novel wound dressings owing to the unique properties of constituent units. Activating such dressings with light at the sight of a wound provides additional therapeutic opportunities. This narrative review summarizes the landscape of light‐activated biomaterials used as wound dressings, with special emphasis on studies demonstrating efficacy in preclinical wound healing models.

## 1 INTRODUCTION

Cutaneous wounds are a major medical and financial burden on the global level, with nearly 6 million chronic wound cases costing more than $20 billion per year in the USA annually.^1^ Complications to wound healing occur in several common comorbidities, such as infection, advanced age, and diabetes. For example, diabetic foot ulcers leading to lower limb amputations, for which diabetic patients have a 15% lifetime risk, have a 5‐year survival rate of only 20%–30%—on par with the overall cancer survival rate.^2^, ^3^ Wound healing is a highly complex process involving the orchestration of immune, vascular, stromal, and parenchymal cells of the skin across a dynamic temporal cascade of overlapping phases generally classified as (i) hemostasis, (ii) inflammation, (iii) proliferation, and (iv) remodeling.^4^, ^5^, ^6^ Any delay in the orderly progression of events in the spectrum of wound healing phases can result in chronicity and increased morbidity and mortality. Specific pharmacologic agents that can modify wound healing are under investigation to target inflammation, fibrosis, and angiogenesis. Still, clinical translation has failed in nearly all cases, and the only FDA‐approved biologic for wound healing (becaplermin) is only partially effective in some cases and comes with a black box warning contraindicating its use in patients with cancer.^7^, ^8^, ^9^ By comparison, the development of novel wound dressings has resulted in a rapidly booming market of clinically viable products.^10^

Wounds are perhaps the oldest disease known to humans, with evidence of wounds discernable even on the fossils of *Australopithecus africanus* from about 2 to 3 million years ago.^11^ The oldest medical treatise known is a clay tablet dating to approximately 2100 bce that indicates three “healing gestures”—washing the wounds, making the plasters (herbs, ointments, and oils), and bandaging the wound.^12^ Bandages are likely as old as clothing itself, and the oldest known bandages in the ancient world were strips of cloth applied with splints found in Egypt,^11^ while in the New World, the oldest bandage was found in Peru from approximately 1500 years ago consisting of a 1‐in. thick cotton roll which would have been wound around an injury and held in place by strands of a woolen cord.^13^ The “moist wound healing theory” proposed by Professor George D. Winter in 1962 revolutionized the field of modern wound care when he demonstrated in seminal studies that a moist wound environment (mediated by coverage with a polythene film) was key for appropriate epithelialization and closure of wounds in young pigs^14^ while contemporary studies by Piskozub evaluated the idea that wound dressings protected against secondary microbial infection.^15^

Another key advancement in the 1960s was the first report of crosslinked synthetic hydrogels for use in biomedical applications by Wichterle and Lim, which set the stage for a new generation of wound dressing engineering.^16^ The use of naturally derived biopolymer constituents has become an increasingly popular strategy over the past 50 years with recent advancements reviewed recently by several groups.^17^, ^18^, ^19^ One modern approach for generating such biomaterial dressings is photoinitiated polymerization or curing of functionalized scaffold components, which can be done either at the bench for preassembled fabrication or in situ within the wound environment.^20^ While there are hundreds of studies on photocrosslinked biomaterials for potential use as wound dressings, few describe in situ generation of the dressing, and even fewer have been demonstrated in animal models of dermal wounds.^21^ Here, we present an updated, landscaping narrative review of light‐activated biomaterials developed for use in wound healing that have been demonstrated to be both in situ activated or generated and which have been demonstrated with in vivo efficacy in animal models. Materials that have been fabricated at the bench for use in animal models were not included, nor were materials that were only validated for cytocompatibility in vitro or with exclusively ex vivo testing. We have organized the review by categorizing based on the photo‐responsive elements utilized to generate the wound dressings across ultraviolet (UV), visible, and near‐infrared (NIR) irradiation regimes to emphasize the variety of ways in which a single type of photosensitizing agent can be adapted to develop therapeutic dressings in situ.

## 2 CONSIDERATIONS WITH WAVELENGTH SELECTION

**FIGURE 1:**
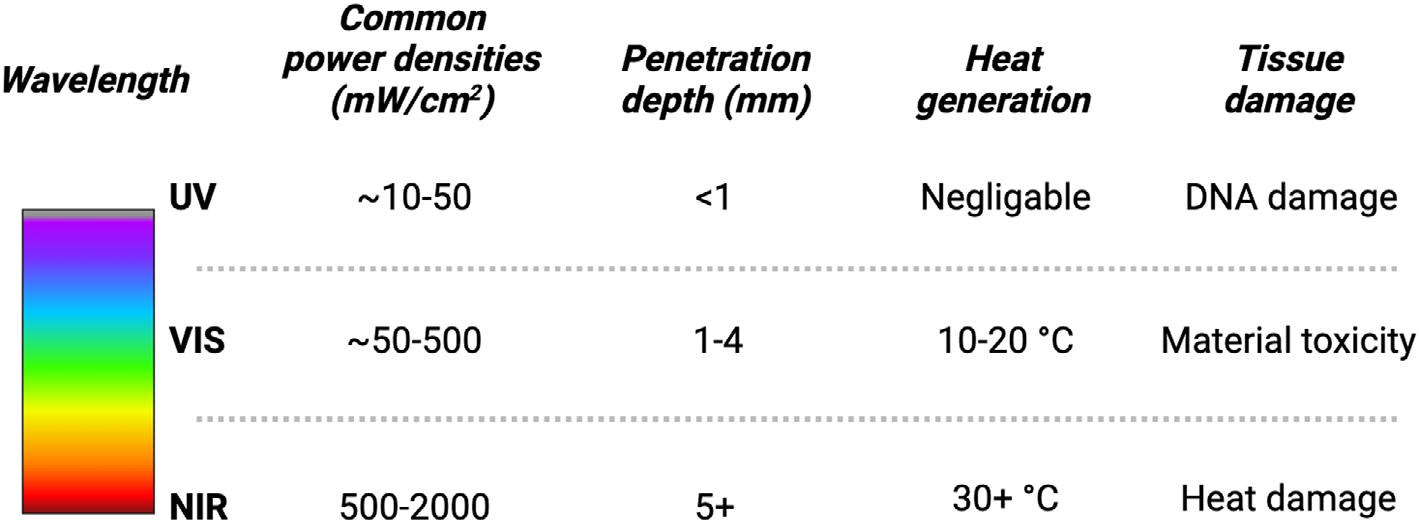
Overview of useful parameters to consider when selecting an activation spectrum for light‐activated biomaterials for tissue repair.

The choice of activating wavelength is a crucial determinant in strategizing the development of light‐activate biomaterials for wound healing applications (Figure 1). Factors such as absorption and scattering by the dressing material or adjacent skin can dramatically affect available light power for initiating polymerization chemistries or facilitating heat‐induced crosslinking.^22^ While UV light can induce convenient crosslinking chemistries at relatively low powers, penetration depths are limited to several tens or hundreds of microns.^22^ Conversely, NIR light can penetrate several millimeters into tissue but often requires higher power densities.^23^ Where available in the literature, we have noted in the following sections the power densities used for in vivo application of the presented light‐activate materials to give context to this consideration. Generally, UV‐activated materials are activated with power densities in the range of tens of milliwatts per square centimeter, while visible light chemistries are commonly in the hundreds of milliwatts per square centimeter, and NIR chemistries are reported in the multiple watts per square centimeter range. Accordingly, heat effects may also be essential to consider. While penetration depth is inversely proportional to wavelength, heat generation is directly proportional to photocuring wavelength with UV producing negligible heat,^24^ visible light generally producing several degrees centigrade heat generation depending on the substrate,^25^ and NIR chemistries producing sufficient heat to be considered a photothermally induced process.^26^ Indeed, with some modalities of photothermal activation of biomaterials using NIR heat, zones of tissue damage have been characterized,^27^ and while these are generally nonsignificant concerning overall healing properties, additional tissue damage due to heat‐generating wavelengths should be evaluated. Likewise, while UV‐induced photoinitiation results in low heat generation at low power densities, exposure to UV light can damage DNA with potential negative downstream effects.^28^ Finally, while the wavelengths used for visible‐light photocrosslinking are generally safe, the materials and photoinitiators may exhibit toxicity.^29^

## 3 UV LIGHT‐ACTIVATED MATERIALS

UV light is categorized in the ranges of UVA (315–400 nm), UVB (280–315 nm), and UVC (100–280 nm). While UV light can damage cells and tissues via mutagenic mechanisms involving DNA, the high energy of UV light can facilitate a variety of photochemical reactions at relatively low power densities compared to longer wavelength light. Accordingly, UV‐mediated chemistries have garnered much attention for in situ wound healing biomaterials, particularly with the convenience of direct conjugation of side groups to hydrogel constitutions (e.g., methacrylation). The following section summarizes the most common photonic chemistries for in situ UV‐activated biomaterials (Table 1).

**TABLE 1:** UV light sensitizers.

Agent	Structure examples	Properties	References
Lithium phenyl‐2,4,6‐trimethylbenzoylphosphinate (LAP)	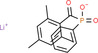	Long UV and visible absorption. Crosslinks methacrylates.	22, 23, 24, 25, 26, 27, 28, 29, 30, 31, 32
2‐Hydroxy‐1‐(4‐(2‐hydroxyethoxy)phenyl)‐2‐methylpropan‐1‐one (Irgacure 2959)	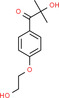	Wide UV absorption. Crosslinks methacrylates. FDA approved.	33, 34, 35, 36, 37, 38, 39, 40
*o*‐Nitrobenzene	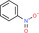	Wide UV absorption. Can be grafted to other molecules for intrinsic crosslinking.	41, 42, 43, 44, 45, 46
Azide and lactobionic acid	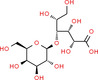	Wide UV absorption. Can be grafted to other molecules for intrinsic crosslinking.	47
Methylene blue	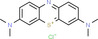	UV and far‐red absorption. FDA approved.	48
Afterglow luminescent particles (ZnS:Ag,Co)	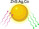	UV absorption. Long‐duration luminescence in visible spectrum.	49
Styrylpyridinium	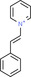	UV absorption with multiple derivatives absorbing in visible spectrum. Can be grafted to other molecules for intrinsic crosslinking.	50

### 3.1 Lithium phenyl‐2,4,6‐trimethylbenzoylphosphinate for UV

Lithium phenyl‐2,4,6‐trimethylbenzoylphosphinate (LAP) is a water‐soluble long UV and violet light‐excitable Type I photoinitiator used in a variety of biomedical applications and thought to be cytocompatible,^30^ though some reports indicate the potential for cytotoxicity upon irradiation.^31^ LAP is one of the most common photoinitiators for facilitating crosslinking chemistry between methacrylated molecules. Kim et al. reported the development of a rapid photocuring sealant based on methacrylated silk fibroin (Sil‐MAS) initiated by LAP (0.3% wt/vol), which they tested in several in vivo and ex vivo systems.^32^ They photocured their Sil‐MAS sealant in situ in incisional wounds in Sprague Dawley rats using 365‐nm UV light for 10 s and compared it with a gauze‐covered and collagen‐based hemostatic dressing. Faster closure was observed by 7 days postinjury, and molecular analysis or protein levels indicated a more rapid proliferative (cyclin D1), angiogenic (vascular endothelial growth factor [VEGF]), and epithelial‐to‐mesenchymal (fibronectin and vimentin) response versus gauze and hemostatic dressing, supporting a more robust reparative biologic response with Sil‐MAS.^32^ Similarly, Pang et al. utilized methacrylated silk fibroin (in this study referred to as SF‐MA) and methacrylated borosilicate in the presence of LAP (0.2% wt/vol) as a proangiogenic wound dressing.^33^ The authors tested their material in a streptozotocin‐induced diabetic‐impaired wound healing model in Sprague Dawley rats with 30 s of in situ UV photoactivation. The authors observed accelerated wound closure, enhanced angiogenesis, and increased collagen deposition with SF‐MA alone and, enhanced further in the presence of borosilicate.^33^ Further investigation supported a proresolution immune phenotype, with proportionate reductions in CD86 proinflammatory macrophages and increases in CD206 proresolution macrophages at 3, 10, and 21 days postinjury. Interestingly, inducible nitric oxide synthase and transforming growth factor‐β (TGF‐β) levels were enhanced on Day 3 with the SF‐MA‐based dressings, indicating a boosted early inflammatory component of healing, which is known to stimulate faster closure.^33^ A methacrylated hyaluronic acid hydrogel with 0.1% wt/vol LAP differentially loaded with amnion‐derived conditioned medium containing additional nitrobenzene groups to facilitate further photo‐induced tissue bonding was described by Zhang et al., which they tested in a C57BL/KsJ db/db diabetic impaired wound healing model.^34^ One minute of 365‐nm photocuring at 50 mW/cm^2^ was used for in situ photoactivation in non‐splinted full‐thickness wounds. The authors observed a significant acceleration of wound closure with the hydrogel alone, which was further enhanced by the inclusion of the conditioned medium.^34^ Hydrogel and conditioned medium application were additionally associated with an increased proresolution macrophage phenotype and enhanced angiogenesis.^34^

The most common methacrylated constituent of crosslinked wound dressing biomaterials is gelatin methacrylate (GelMA). Several groups have reported in situ photocuring of GelMA‐based dressings using LAP chemistries. Nuutila et al. developed a handheld bioprinter employing a 1 W 395‐nm light‐emitting diode (LED) to “print” VEGF‐loaded GelMA hydrogel (0.067% wt/vol LAP) in situ in full‐thickness wounds in pigs.^35^ The authors observed enhanced wound closure with topical VEGF and empty printed GelMA, which was further enhanced by releasing the VEGF from printed GelMA hydrogel scaffolds in situ.^35^ These enhancements were associated with improvements in the number of Rete ridges, scar quality, and angiogenesis.^35^ Similarly, Zhou et al. developed a UV‐exciting in situ handheld bioprinter to instill a 0.5% wt/vol LAP‐initiated GelMA dressing with copper‐containing bioactive glass nanoparticles in streptozotocin‐induced diabetic Sprague Dawley rats.^36^ They observed faster closure rates versus GelMA alone, associated with improved proresolution immune phenotypes, collagen deposition, and angiogenesis.^36^ Yu et al. performed in situ photocuring with 365 nm (9 W) for 1 min in a Sprague Dawley full‐thickness rat wound healing model with GelMA‐based hydrogels (1% wt/vol LAP) containing various amounts of thioglycolic acid‐modified chitosan and 3‐buten‐1‐amine‐modified polycaprolactone nanofibers and observed composition‐dependent acceleration of wound healing with modified chitosan and nanofibers improving healing versus GelMA alone.^37^ The same group recently reported an additional modification wherein the hydrogel platform was additionally supplemented with a decellularization product of dermal extracellular matrix‐methacryloyl/poly (ethylene glycol) diacrylate (ddECMMA/‐PEGDA) with 0.1% wt/vol LAP, which they observed further enhanced wound closure rates and collagen deposition in full‐thickness wounds in Sprague Dawley rats with in situ photocuring (365 nm, 9 W, 60 s).^38^ Xie et al. modified GelMA hydrogels (0.25% wt/vol LAP) with a hyperbranched terpolymer containing imidazole groups to coordinate ascorbyl palmitate (AP) nanosheets and silver ions and observed enhanced wound closure with the combination of AP and silver ions with associated pro‐resolution chemokine phenotypes when tested in full‐thickness wounds in Sprague Dawley rats with 1 min of in situ photoactivation with 365‐nm light.^39^ Interestingly, the authors described that rapid dressing changes could be performed by spraying 4°C Cu(NO_3_)_2_ solution on the dressings, causing a rapid release from tissue bonding.^39^ Recently, Balaji et al. reported a GelMA composite (0.3% wt/vol LAP) loaded with lignin‐based nanoparticles with or without oxygen‐producing calcium peroxide and tested efficacy in a full‐thickness splinted wound healing model in C57BL/6N mice with in situ crosslinking using a high‐intensity UV flood lamp for 30 s.^40^ The authors observed that the introduction of the complete formulation of calcium peroxide‐containing lignin‐modified hydrogels resulted in enhanced wound granulation with improved infiltration of alpha‐smooth muscle actin‐positive cells at 7 days postsurgery and increased blood vessel formation.^40^

### 3.2 2‐Hydroxy‐1‐(4‐(2‐hydroxyethoxy)phenyl)‐2‐methylpropan‐1‐one (Irgacure 2959)

**FIGURE 2:**
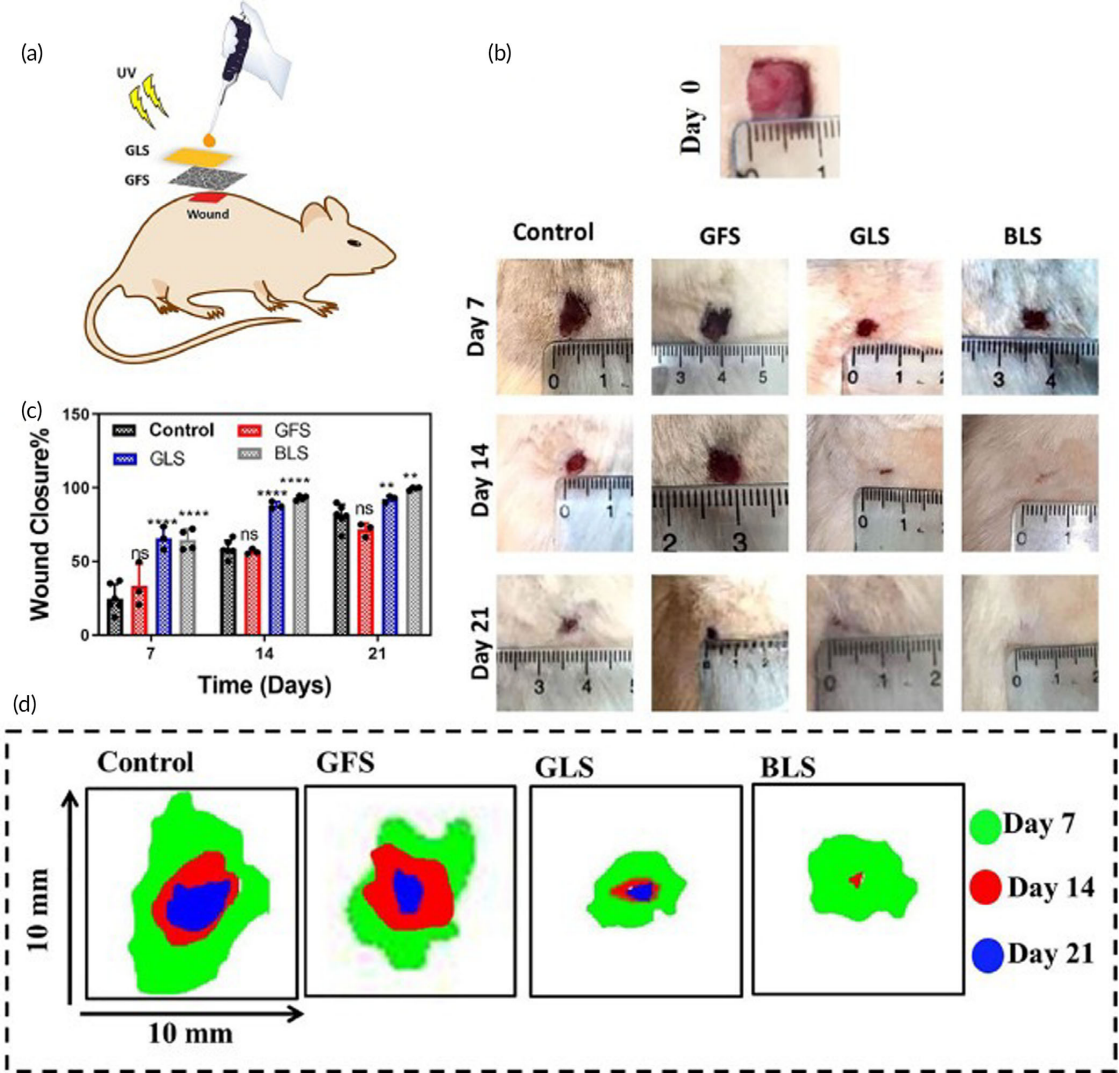
In vivo evaluation of biomimetic nanoengineered scaffold in rats. (a) Schematic representation of in situ deposition and activation of 100 μL of Irgacure 2959‐photoinitiated precursor solutions directly into the wound bed with activation using an OmniCure S200 UV light source at a power of 6.9 mW/cm^2^ for 60 s. (b) Gross evaluation and (c) planimetric quantification of wound closure. (d) Comparative wound closure mapping of various wound dressing formulations at different time points. Reprinted from Zandi et al.^48^

Irgacure 2959 is one of the few FDA‐approved photoinitiators for use in human biomedical applications, including in dental grafts.^41^ Tang et al. investigated a methacrylated silk fibroin hydrogel functionalized with chlorine e6 photoinitiated by Irgacure 2959 (0.5% wt/vol) in situ with 365‐nm light in a *Staphylococcus aureus*‐infected full‐thickness wound healing model in Balb/c mice and observed Ce6‐functionalized hydrogels resulted in 3‐log reduction in bacterial load, faster wound closure, and reduced scar appearance versus silk hydrogels alone or controls.^42^ Wang et al. developed a self‐healing hydrogel composed of methacrylated PLGA and cyclodextrin‐conjugated PLGA photoinitiated by Irgacure 2959 (5% of formulation weight). The crosslinked hydrogels were tested in an incisional wound healing model in Sprague Dawley rats with in situ activation by 365‐nm light (20 mW/cm^2^, 10 min).^43^ The authors observed similar wound closure to sutures with better cosmesis, with no “railroad tracks” observed in hydrogel‐sealed incisions.^43^ Xu et al. investigated a composite hydrogel containing methacrylated quaternary ammonium chitosan, polyvinylpyrrolidone, and dopamine (DOPA) photoinitiated by 0.7% wt/vol Irgacure 2959 and tested its efficacy with multifunctional DOPA‐mediated NIR‐induced photothermal microbial clearance in an *S. aureus*‐infected full‐thickness wound model in Balb/c mice with in situ photocuring with irradiation at 365 nm (260 mW/cm^2^) for 15 s.^44^ Interestingly, the hydrogel with or without NIR irradiation resulted in enhanced wound closure with only slight enhancement by photothermal activation, demonstrating the intrinsic therapeutic efficacy of the hydrogel alone.^44^ Skardal et al. successfully produced hydrogels without methacrylate moieties by using thiolated hyaluronic acid, heparin, and gelatin constituents crosslinked with PEGDA initiated by Irgacure 2959 (2% wt/vol) to deliver amniotic fluid‐derived stem cells for wound repair via in situ bioprinting.^45^ When tested in athymic nu/nu mice with 365 nm in situ crosslinking, heparin‐containing hydrogels facilitated faster wound closure versus hyaluronic acid hydrogels alone, demonstrating a therapeutic effect of retained cytokines secreted by the embedded stem cells.^45^ Chen et al. incorporated FDA‐approved desferrioxamine copper chelating agents in GelMA hydrogels (0.5% wt/vol Irgacure 2959) and tested their efficacy in streptozotocin‐induced diabetic impaired wounds in Sprague Dawley rats with in situ activation at 6.9 mW/cm^2^ UV light (360–480 nm) for 1 min.^46^ The investigators observed accelerated wound closure with desferrioxamine cargo versus GelMA alone, with concomitant expression of VEGF and hypoxia‐inducible factor‐1α and generation of significantly more blood vessels.^46^ Similarly, Augustine et al. incorporated structural stable poly(3‐hydroxybutyrate‐*co*‐3‐hydroxyvalerate) and EGF in GelMA matrices with 0.5% wt/vol Irgacure 2959 and observed EGF release‐induced acceleration of wound closure in streptozotocin‐induced diabetic impaired wounds in Sprague Dawley rats with in situ photocuring with a handheld dental UV crosslinker for 3 min.^47^ Zandi et al. utilized gelatin nanofibers (GFS) with a GelMA‐laponite photocrosslinkable layer using Irgacure 2959 (concentration not reported) to develop a bilayer composite dressing loaded with EGF.^48^ While the nanofibers acted as a dermis‐like layer, the photocrosslinked layer acted as an epidermis‐like layer. Wister albino rats were used in a non‐splinted full‐thickness wound healing model and treated with GFS alone, crosslinked hydrogel layer alone (GLS), or the GFS combined with the GLS to generate bilayer scaffold (BLS) with crosslinking in situ at 365 nm for 60 s at 6.9 mW/cm^2^ (Figure 2). The authors observed significant early closure with GLS and BLS that was maintained over 21 days of follow‐up.^48^ In comparison, GFS alone did not accelerate healing, which was accompanied by proportional improvements in collagen deposition and architectural remodeling.^48^

### 3.3 *o*‐Nitrobenzene

Several nitrogen‐containing benzene moieties have been described as amenable to direct conjugation to hydrogel constituents, enabling direct photoinitiation without the addition of accessory photosensitizers. The most common of these groups is *o*‐nitrobenzene, which forms imine crosslinks of aldehyde and hydrazine upon photoinitiation. Yang et al. first demonstrated the conjugation of *o*‐nitrobenzene to hyaluronic acid (2.5%–5% wt/vol) for in situ photocuring and tissue bonding in wounds and observed accelerated wound closure by mechanical stimulation in a full‐thickness wound healing model in Sprague Dawley rats when activated with 365‐nm LED light (20 mW/cm^2^) for 3 min.^49^ Similarly, Bo et al. conjugated *o*‐nitrobenzene to hyaluronic acid (8% wt/vol) and developed a hydrogel in complex with decellularized dermal matrix capable of being loaded with adipose‐derived stem cells (ASCs).^50^ The authors performed a full‐thickness wound model in athymic nude mice with in situ photocuring at 365 nm (10 mW/cm^2^) for 90 s and observed that while ASCs or the hydrogel alone accelerated wound closure, the combination of ASCs within the HA hydrogel facilitated the fastest closure, accompanied by proportional effects on collagen deposition and neoangenesis.^50^ Zhao et al. likewise used *o*‐nitrobenzene conjugation with hyaluronic acid and cyclodextrin (3.7% wt/vol) to develop an EGF‐releasing supramolecular hydrogel dressing and tested both non‐irradiated and UV‐irradiated (365 nm, 50 mW/cm^2^, 15 min) treatment in a full‐thickness wound healing model in rats.^51^ UV irradiation led to the fastest wound closure and stimulated the greatest intrinsic expression of TGF‐β1 and angiogenesis at Days 4 and 10 postinjury.^51^ In another example, Zhang et al. used *o*‐nitrobenzene conjugated hyaluronic acid hydrogels to deliver *o*‐nitrobenzene‐conjugated PLGA (6.7% wt/vol) microcapsules loaded with TGF‐β1 to full‐thickness wounds in CD1 mice with in situ activation at 365‐nm (20 mW/cm^2^ for 3 min) and observed enhanced wound closure with microcapsules containing TGF‐β versus microcapsules alone.^52^ The authors further investigated their material in a porcine full‐thickness wound model in Yorkshire pigs with the same photoactivation parameters and observed the same effect as well as improvements in scar elevation index.^52^ Ma et al. conjugated *o*‐nitrobenzene to carboxymethyl chitosan (CMC) (6% wt/vol) and evaluated different proportions of conjugated and non‐conjugated CMC in a full‐thickness wound healing model in Balb/c mice with in situ activation with 320–500 nm light (30 mW/cm^2^) for 60 s, and found significantly accelerated closure with improved angiogenesis in formulations containing higher amounts of conjugated CMC.^53^ Combining the prior techniques, Mao et al. developed a dual‐network hydrogel consisting of azide‐conjugated carboxymethyl chitosan (CMC‐AZ) and *o*‐nitrobenzyl hyaluronic acid (HA‐NB; 2%–3% wt/vol) with or without loaded amoxicillin.^54^ When tested in a full‐thickness wound healing model in ICR mice gelled in situ by UV irradiation, the authors observed a formulation‐dependent acceleration of wound closure with CMC‐AZ and HA‐NB loaded with amoxicillin demonstrating the greatest effect versus hydrogels without drug or without azide conjugation of CMC, associated with proportional increases in angiogenesis, improvements in collagen deposition, and reduction in inflammation.^54^

### 3.4 Other UV photosensitizers

Miscellaneous other photosensitizers have been used to enable UV‐activated curing in situ. Ishihara et al. generated a photoinitiator‐independent, self‐crosslinking azide and lactobioinic acid chitosan (1%–3% wt/vol) for use in wound occlusion.^55^ In situ UV irradiation for 90 s in a full‐thickness mouse wound model significantly accelerated closure during the first 7–10 days of healing versus wounds without irradiation or control, untreated wounds.^55^ Methylene blue (0.0056% wt/vol) was used as photoinitiator by Chang et al. to generate a hydrogel composed of gelatin, 3,3′,4,4′‐benzophenone tetracarboxylic dianhydride, and 2‐hydroxethyl methacrylate (HEMA) along with chitosan and poly(ethylene glycol) diacrylate (PEGDA). The authors irradiated a polymer pre‐solution with UV for 20 s in full‐thickness wounds in mice and significantly enhanced wound closure versus control, untreated wounds.^56^ Kim et al. developed a complex two‐phase system in which UV‐absorbing controlled afterglow luminescent particles (ALPs) of ZnS:Ag,Co (8 mg/mL), in turn, excited Rose Bengal‐conjugated hyaluronic acid (HA‐RB, 500 μM) to directly crosslink collagen in wounds via photochemical bonding.^57^ When tested in a deep tissue incision wound model in Balb/c mice with an on/off cycle of 30 s for 15 min with a UV lamp or green light, ZnS:Ag,Co‐containing HA‐RB materials activated with UV light facilitated the greatest healing and recovery of skin tensile strength versus sutures (control skin ~40 kPa, sutures ~57 kPa, and UV light‐illuminated ALPs ~78 kPa), HA‐RB illuminated with green light directly, or HA‐RB containing ZnS:Ag,Co without illumination.^57^ Recently, Zhang et al. reported the direct conjugation of the styrylpyridinium small molecule photoinitiator dye to polyvinyl alcohol (PVA) (15% wt/vol) to develop a photocuring, injectable hydrogel in complex with oxidized sodium alginate.^58^ The material was evaluated in an *S. aureus*‐infected full‐thickness wound model in mice with in situ photoactivation of dressing‐to‐wound interaction at 365 nm for 30 s. Subsequent differential irradiation of incorporated polydopamine with 808‐nm light for 10 min demonstrated a significant acceleration of wound closure with the hydrogel alone that was further enhanced by NIR stimulation, accompanied by enhanced angiogenesis and collagen deposition in proportion to the healing efficacy.^58^

## 4 VISIBLE LIGHT‐ACTIVATED MATERIALS

The visible light spectrum includes 400 nm to about 750 nm. Different wavelengths of visible light have been used as a curing energy source to crosslink biomaterials at specific functional sites, which led to form a hydrogel by harnessing chemistries enabled by complex photosensitizers. Versus UV irradiation, visible light is significantly less likely to cause mutagenic damage to tissue, making it an attractive domain within which to activate biomaterials, while also requiring reasonably low power densities. The following section summarizes the most common agents mediating visible light activation of biomaterials for wound healing (Table 2).

**TABLE 2:** Visible light sensitizers.

Agent	Structure examples	Properties	References
Ruthenium complexes	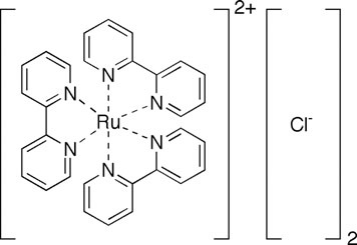	Blue light absorption with red light emission.	52, 53, 54, 55, 56, 57
Eosin Y	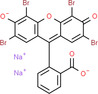	Blue–green light absorption.	59, 60
Riboflavin	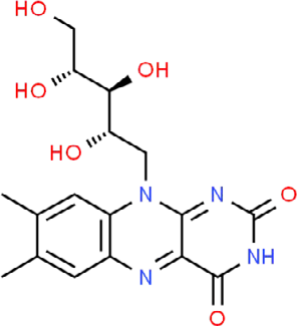	Blue light absorption. Endogenous protein with high biocompatibility.	61, 62
Lithium phenyl‐2,4,6‐trimethylbenzoylphosphinate (LAP)	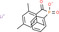	Long UV and visible absorption. Crosslinks methacrylates.	63, 64, 65, 66, 67
Rose Bengal	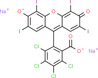	Green light absorption. FDA approved.	68, 69, 70
Metal nanoparticles	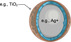	Tunable absorption depending on formulation.	71, 72

### 4.1 Ruthenium metal complexes

Ruthenium is a Group VIII transition metal with two primary oxidation states, Ru(II) and Ru(III). Coordinated ruthenium complexes have found biomedical use as a photosensitizer for photodynamic therapy and drug delivery.^73^ Incorporation of ruthenium complexes into biomaterials can imbue photo‐activatable properties under certain formulations. For example, fibrin glue is composed primarily of fibrinogen and cofactors and is a common, clinically used tissue adhesive. Despite its widespread use, fibrin glue exhibits negative properties, including long curing times, low bond strength with tissue, and complex, costly, and potentially unstable formulations. In an effort to address these issues, Elvin et al. developed a formulation of fibrinogen mixed with Ruthenium trisbipyridyl chloride (Ru(II)(bpy)_3_Cl_2_) (2 mM) and the oxidizing agent sodium persulfate (SPS), which form dityrosine covalent bonds in adjacent proteins upon white light illumination.^59^ This mixture was applied to rat skin incisions and cured with a 600‐W tungsten halide lamp for 20 s at a distance of 150 mm. Histologic analysis showed reduced inflammation and evidence of collagen deposition within 1 week of wounding with both the photocrosslinked fibrinogen and commercial fibrin glue, and tensile strength recovery was similarly improved with both formulations from 1 to 10 weeks postinjury, both having approximately 120% native skin strength versus only 80% with wound clips.^59^ Importantly, photocrosslinking fibrinogen obviated the need for thrombin or other components of the clotting cascade, simplifying the overall formulation without loss in efficacy. Jeon et al. utilized similar chemistries with mussel adhesion protein (MAP), which allows mussels to adhere in wet environments.^60^ The group developed a light‐activated mussel protein‐based bioadhesive (LAMBA) composed of a recombinant MAP containing ~20 mol% tyrosine content to increase dityrosine formation and improve overall structural stability with 2 mM Ru(II)(bpy)_3_Cl_2_ and persulfate catalysts and evaluated its functionality as surgical glue. LAMBA exhibited rapid (<60 s) room temperature crosslinking with a blue (460 nm) dental curing lamp at 1200 mW/cm^2^ at 20 mm.^60^ When tested against sutures, fibrin glue or cyanoacrylate glue in an incisional wound model in Sprague Dawley rats, in situ application of LAMBA resulted in a nearly twofold increase in skin breaking strength at 2 weeks postinjury (sutures ~620 kPa, fibrin glue ~510 kPa, cyanoacrylate ~480 kPa, LAMBA ~1000 kPa).^60^ The same group subsequently further engineered their system, now with 50 mM Ru(II)bpy^2+^_3_, to incorporate collagen‐binding domains into the MAP (MAP‐mCPR), facilitating interaction between MAP and collagen I in the skin.^61^ When tested in a non‐splinted excisional wound healing model in Sprague Dawley rats, LAMBA with MAP‐mCPR demonstrated enhanced scar reduction and improved collagen remodeling versus MAP without collagen‐binding properties, which was further improved by the addition of a dermatan sulfate cofactor.^61^ Jeon and colleagues thus demonstrated with LAMBA the potential for engineering biomimetic and bioinspired architectural and targeting design for in situ photoactivable materials in both incisional and excisional wounds. Recently, Zhu et al. used the same Ru(II)(bpy)_3_Cl_2_ (0.2–2 mM) and SPS photochemistry to develop a first‐aid bioadhesive using the tyrosine‐rich milk protein casein.^62^ Using casein for such biomaterials provides practical advantages of being naturally derived (non‐recombinant) with pre‐existing multi‐million‐ton production pipelines servicing the global dairy, papermaking, leathermaking, construction, and plastics industry annually.^63^ Using an ordinary handheld flashlight, the authors described gelation within 10 s in situ in a full‐thickness non‐splinted wound healing model in mice and resultant improvements in early wound closure rates at Day 4 and Day 8, increases in collagen content, and shifts in the immunologic profile at the cytokine and cellular level to a more proresolution state.^62^ Kushibiki et al. utilized an alternative ruthenium photosensitizer, pentamethylcyclopentadienyl triphenylphosphine ruthenium chloride (Cp*RuCl(PPh_3_)_2_) (0.25–1 mM) with SPS to photocrosslink gelatin in situ using a blue 455 nm LED (30 mW/cm^2^) for 30 s in both a full‐thickness non‐splinted wound model in diabetic C57BLKS/J Iar‐+Lepr^db^/+Lepr^db^ and in a skin flap survival model in genetically modified mice expressing the ATP sensor ATeam.^64^ The authors further demonstrated the loading and release of basic fibroblast growth factor (bFGF), which normally suffers from a very short half‐life but nonetheless has been used with varying success in clinical settings.^65^, ^66^ In their studies, the authors report the hydrogels alone demonstrated enhanced wound closure, granulation, and angiogenesis versus saline treatment and bFGF direct treatment, which was further enhanced by loading and releasing bFGF from the hydrogels in the full‐thickness wound model.^64^ The skin flap survival model showed improved angiogenesis and preservation of ATP levels within the flap, indicating sustained tissue survival, both of which were again enhanced by loading and releasing bFGF from the hydrogel.^64^

### 4.2 Eosin Y

While Eosin Y is commonly used as a dye in histology, it exhibits strong visible light absorption, making it appropriate as a photosensitizer for biomaterial fabrication. Photocrosslinking can be achieved through a free radical polymerization reaction using the common triarylmethane histologic dye Eosin Y as a photosensitizer, triethanolamine (TEA) as a co‐initiator, and the comonomer *N*‐vinylcaprolactam with irradiation using visible light in the range of 430–530 nm. Guo et al. describe a hemostatic adhesive (HAD) composed of GelMA and snake venom‐derived reptilase (a hemocoagulase) photoinitiated by 0.5 mM Eosin Y to provide prohealing wound adhesion as well as hemostatic control. Illumination of HAD for 1 min in situ in an incisional wound model in Sprague Dawley rats demonstrated superior wound approximation versus fibrin glue or GelMA alone and similar granulation, tissue bridging, and healing profiles to sutures at Day 5 and Day 20 postinjury.^67^ Using the same photoinitiator (Eosin Y, 0.1–0.5 mM) and co‐initiator (TEA) along with 1% vol/vol vitamin C and white light activation, Wang et al. reported the development of a hydrogel passively loaded with an antibacterial peptide (CM11: WKLFKKILKVL) and simultaneously crosslinked and functionalized with a vascular endothelial growth factor mimetic peptide (KLT: KLTWQELYQLKYKGI) containing a Gly–Cys–Gly modification at the N‐terminal with a thiol‐ene reaction.^74^ In situ gelation occurred in less than a minute with high powered (1200–2000 mW/cm^2^) laser illumination of the hydrogel in the range of 420–480 nm. When tested in a full‐thickness methicillin‐resistant *S. aureus* (MRSA)‐infected wound model in Balb/c mice, empty hydrogels exhibited faster closure versus untreated wounds alone, with further acceleration using either CM11‐formulated or KLT‐formulated hydrogels, and the fastest wound closure with the combination CM11/KLT‐formulated hydrogels.^74^ These trends were, respectively, supported by enhancements in collagen deposition and angiogenesis and reduced inflammation.^74^

### 4.3 Riboflavin

Riboflavin or vitamin B_2_ is naturally derived and absorbs visible light in the range of 220–450 nm and can act as an alternative to synthetic photoinitiators, though a co‐initiator is needed as a proton donor to initiate the polymerization reaction.^68^ Jin et al. reported the development of an antibacterial wound dressing utilizing a visible light‐cured methacrylated collagen (ColMA) hydrogel blended with a complex of 2‐hydroxypropyl‐beta‐cyclodextrin (HP‐β‐CD) and triclosan (TCS) (CD‐ic‐TCS).^69^ This complex was photoactivated using blue light (430–485 nm) for 10 s in situ, facilitated by riboflavin 5′‐monophosphate sodium salt (riboflavin, 12 μM) as a photoinitiator in a non‐splinted full‐thickness wound model in Balb/c mice. Mice treated with hydrogels containing the CD‐ic‐TCS complex exhibited accelerated wound closure at Days 4 and 7 posthealing, though overall closure between groups was not substantially different by Day 10 or 14 postwounding, which may attributable to wound contraction due to primary intention healing in the absence of splints.^69^ Despite the similar overall planimetric wound closure profile, histologic evidence of scar formation was reduced with in situ hydrogels containing CD‐ic‐TCS or TCS alone versus hydrogel and commercially available hydrocolloid dressing.^69^

### 4.4 LAP for visible light

**FIGURE 3:**
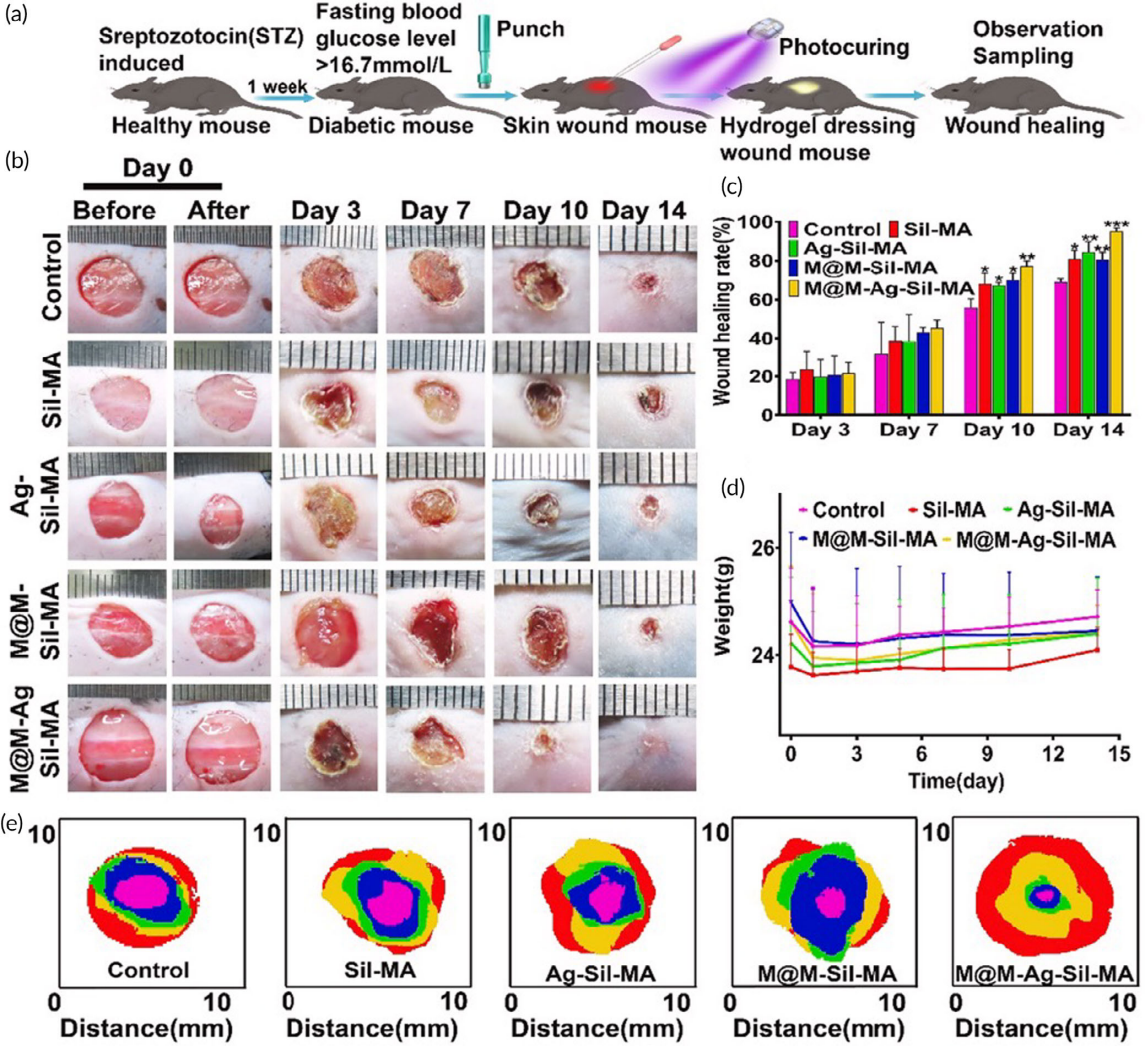
Evaluation of in situ violet light‐activated silk dressing in an induced diabetic mouse model. (a) Schematic representation of streptozotocin‐induced diabetes with full‐thickness wound generation and in situ activation of a silk‐based wound dressing using 405‐nm light for 25 s using LAP‐based photoinitiation. (b) Gross evaluation and (c) planimetric quantification of wound closure. (d) Weight tracking over the course of the experiment. (e) Comparative wound closure mapping of various treatments over time. Reprinted from Mei et al.^75^

As mentioned above, LAP chemistries are also compatible with visible light sensitization. Wang and colleagues developed a TCS‐grafted gelatin and GelMA composite biomaterial (TGM) crosslinked by LAP (0.2% wt/vol) with 405 nm violet–blue light.^70^ The authors describe the material as having moisturizing properties and demonstrated antimicrobial efficacy against a variety of pathogens in vitro due to the TCS‐grafted component. Interestingly, when tested in vivo in Sprague Dawley rats, in situ photoactivated TGM exhibited a slight but significant acceleration of closure within 14 days of full‐thickness wounding, but gelatin‐GelMA demonstrated no benefit, suggesting TCS grafting provided added benefit.^70^ In another group, Wang et al. adapted LAP chemistries (0.5% wt/vol) to investigate hydrogels with additional adhesive properties by functionalizing the proadhesion molecule DOPA onto GelMA.^71^ The group further modified their hydrogels by loading mesenchymal stem cell extracellular vesicles (EVs) into their hydrogel to impart prohealing angiogenic and wound motility properties. When tested in a full‐thickness wound model in Sprague Dawley rats with streptozotocin‐induced diabetic wound impairment, in situ activated (405 nm, 10 mW/cm^2^ for 1–2 min) GelMA‐DOPA, EVs, or GelMA‐DOPA containing EVs independently and combinatorially accelerated wound closure, reduced wound scar size, reduced inflammation (interleukin‐6) and faster rates of CD31‐positive angiogenic sprouting and pruning over the course of healing.^71^ Tian et al. investigated an alternative composite of GelMA with a modified acryloyl‐(polyethylene glycol)‐elastin (elastin‐PEG‐AC) in the presence of LAP (0.05% wt/vol) and 2 min of 405‐nm light activation to promote full‐thickness wound healing in C57BL6/J mice.^72^ Interestingly, the group explored different formulation ratios for GelMA and elastin‐PEG‐AC and found differential results on wound outcomes. Wound closure and collagen deposition appeared to improve dependent on a higher ratio of elastin‐PEG‐AC over GelMA, while immune and angiogenic responsiveness peaked with an approximately 50:50 ratio of elastin‐PEG‐AC to GelMA.^72^ These findings highlight the difference between mechanical modulation of wound closure with endogenous biological response to hydrogel components. Demonstrating broader utility of LAP outside of GelMA‐based materials, Mei et al. developed a photocurable methacryloxylated silk fibroin (Sil‐MA) hydrogel and further functionalized it by co‐encapsulating metformin‐loaded mesoporous silica microspheres (MET@MSNs or M@M) and silver nanoparticles (AgNPs) photoinitiated by LAP (0.25% wt/vol).^75^ The authors tested their Sil‐MA hydrogel compositions by in situ activation with 405‐nm light for 25 s in C57BL6/J mice with streptozotocin‐induced diabetes with non‐splinted full‐thickness wounds (Figure 3). The authors observed accelerated closure in all Sil‐MA formulations beginning at Day 10 post‐injury. Incorporation of M@M into the Sil‐MA hydrogel facilitated a more proresolution environment beginning at Day 7 postinjury, with a bias toward CD206 macrophages and away from CD86 macrophages, as well as an early resolution of neutrophilic inflammation as seen by a reduction in CitH3‐positive NETosis by Day 3 postinjury. Interestingly, Sil‐MA loaded with M@M was further enhanced by the incorporation of AgNP despite the model not involving a bacterial infection, demonstrating the non‐antimicrobial role of the loaded AgNPs^76^ and potentially revealing low‐level microbial involvement from the cage environment in seemingly normal wound healing.

### 4.5 Rose Bengal

Rose Bengal (4,5,6,7‐tetrachloro‐2′,4′,5′,7′‐tetraiodofluorescein) is xanthene commonly used to interrogate damaged conjunctiva and corneal cells in the eye and as an anticancer photosensitizer.^77^ Na et al. synthesized *O*‐carboxymethyl chitosan (*O*‐CMC) derivatives with furfuryl glycidyl ether (*O*‐CMC/FGE), which formed a highly viscous, non‐flowing hydrogel when illuminated at 420–490 nm for at least 180 s in the presence of 5% wt/vol Rose Bengal.^78^ The authors tested the wound healing efficacy of *O*‐CMC/FGE in a burn model in Sprague Dawley rats, with the material photoactivated in situ for 5 min with or without co‐delivery of epidermal growth factor (EGF). The authors found that *O*‐CMC/FGE accelerated re‐epithelialization and reduced early inflammation with or without EGF co‐delivery. Interestingly, they reported that despite evidence of re‐epithelialization in EGF‐alone or *O*‐CMC/FGE with EGF, collagen maturation was not observed in either group but was observed in the *O*‐CMC/FGE alone group, suggesting a material‐driven prohealing stimulation that could not be further augmented by bioactive co‐stimulation.^78^ In a subsequent study from the same group, Park et al. encapsulated murine epidermal growth factor (mEGF) in the same hydrogel material (with 0.1% wt/vol Rose Bengal) and evaluated efficacy in a non‐splinted full‐thickness wound healing model in Sprague Dawley rats.^79^ The authors found a slight acceleration in epithelialization mediated by the *O*‐CMC/FGE base material, encapsulation with mEGF reduced foreign body reaction to the material and enhanced fibroblast activity in the healing wound. In a complementary study, Heo et al. conjugated furfurylamine to alginate, resulting in furfuryl alginate (F‐alginate), which was combined with 0.5% wt/vol Rose Bengal for visible light reactivity.^80^ Full‐thickness non‐splinted wounds were generated in Sprague Dawley rats, and F‐alginate with or without mEGF was photocured in situ with a dental curing light (420–490 nm) for 7 min. The authors found enhanced epithelialization and fibroblast activity that was further augmented by the incorporation of mEGF, with the F‐alginate acting to limit foreign body responses.

### 4.6 Metal nanoparticles

Metal nanoparticles have received significant attention in recent years due to their intrinsic or functionalizable antimicrobial properties. AgNPs of varying shapes and sizes have been particularly widely characterized due to exhibiting a variety of intrinsic antimicrobial mechanisms, including physical disruption of microbial membranes, biologically induced reactive oxygen and free radical generation, and negative regulation of microbial signal transduction.^81^ Gold nanoparticles, in contrast, are only weakly intrinsically antimicrobial^82^ but can be functionalized to achieve a wide variety of antimicrobial effects.^83^ In addition to reported antimicrobial effects, metal nanoparticles can exhibit visible light‐mediated photothermal effects or photo‐induced reactive species generation, both capable of facilitating in situ generation of biomaterial dressings in wounds. While there are no available in vivo studies to date that utilize metal complexes as in situ photoinitiators or photosensitizers for wound healing and repair, studies that demonstrate the activation of specific properties of these nanoparticle complexes with light in situ have been reported. Wang et al. generated Ag‐doped TiO_2_ (Ag/TiO_2_) nanoparticles incorporated at 0.5% wt/vol into a PVA hydrogel to combat antibiotic‐resistant bacteria by harnessing light‐induced reactive oxygen species (ROS).^84^ The hybrid hydrogel was created through a two‐step process wherein Ag/TiO_2_ nanoparticles were dispersed in PVA and gelled via repeated freeze‐thaw. The hydrogels were evaluated in a non‐splinted full‐thickness wound model in Kumming rats inoculated with *S. aureus*. When exposed to 660‐nm visible light in situ, the hydrogels exhibited potent antimicrobial activity, enabling a near‐double rate of healing versus control wounds, with histologic evidence of reduced inflammation and improved tissue viability at 14 days postinjury.^84^ Huang et al. developed a dual‐responsive system composed of yellow light‐excitable selenium nanoparticles that were further encapsulated with polyethylenimine and modified with sonoreponsive indocyanine green (ICG), which together enabled both photodynamic and sonodynamic therapy modalities.^85^ The multimodal particles were tested in a non‐splinted full‐thickness MRSA‐infected wound healing model in Sprague Dawley rats. After wounding and inoculation with MRSA, the nanoparticles (200 μg/mL) were added topically activated with either 3 min of ultrasound stimulation, 10 min of yellow LED stimulation, or both daily for 13 days. While the nanoparticles alone stimulated improved wound closure versus controls, stimulation with ultrasound, yellow light, or their combination significantly accelerated overall closure rates, with concomitant in matrix remodeling and angiogenesis.^85^ Despite the potent responsiveness of metal nanoparticle complexes, these photosensitizers remain underutilized for in situ biomaterial generation in dermal injuries.

## 5 NIR AND LONGER LIGHT‐ACTIVATED MATERIALS

The wavelength range of NIR light begins just after the visible light range ends (about 700 nm), with subcategorizations placing the NIR I window from 700 to 950 nm and tissue penetration up to approximately 6 mm, and the NIR II window from 1000 to 1700 nm with penetration up to 20 mm.^86^ In practice, light can be produced using either a coherent source (laser) or a noncoherent source such as LEDs. It is regarded as superior in certain biological applications compared to other wavelengths—especially in applications requiring deeper light penetration, such as bioimaging, tumor ablation, and diagnostics. Applications of NIR light with different wavelengths have also been extensively evaluated in wound healing applications. Even though the penetration depth of the light depends on the wavelength and coherence of the incident light, time of irradiation, and the tissue type, both NIR I and NIR II light can penetrate the full thickness of human skin, albeit with the requirement of significantly higher power densities versus UV or visible light.^87^ The effects of NIR irradiation on skin biology can manifest in different forms, such as generation of ROS, initiation of chemical reaction, or modulation of biological pathways and finely tuned photothermal conversion with NIR photosensitizers is commonly employed as an approach to facilitate microbial clearance.^88^ The following describes the most common approaches to promote wound healing using NIR light (Table 3).

**TABLE 3:** NIR light sensitizers.

Agent	Structure examples	Properties	References
Intrinsic protein absorbance	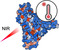	Absorption >1200 nm (NIR II). High biocompatibility.	77, 78, 79, 80, 81, 82, 83
Gold and silver nanoparticles	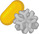	Tunable absorption, primarily at ~808 nm (NIR I).	84, 85, 86, 87, 88, 89, 90
Polydopamine	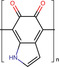	Absorption at 808 nm (NIR I). Naturally derived and facile coating. Intrinsically adhesive.	91, 92, 93, 94, 95
Upconverting nanoparticles	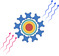	Absorption at 980 nm (NIR I/II) with tunable emission.	96, 97, 98
Metal sulfides (e.g., tungsten sulfide nanosheets)	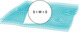	≥808‐nm absorption (NIR I).	99, 100, 101
Cyanine dyes (e.g., indocyanine green)	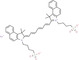	808‐nm absorption (NIR I). FDA approved.	102, 103, 104
Modified carbohydrates	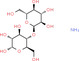	808‐nm absorption (NIR I). Highly biocompatible.	105, 106, 107
Tannic acid	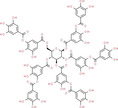	808‐nm absorption (NIR I). Generally regarded as safe by US FDA.	108
MXene	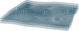	808‐nm absorption (NIR I). Conductive.	109
PEDOT:PSS	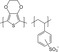	808‐nm absorption (NIR I). Conductive.	110
Carbon nanotubes	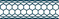	808‐nm absorption (NIR I). Conductive.	111

### 5.1 Intrinsic protein absorbance

Beginning in the 1960s, the earliest examples of laser‐assisted tissue repair (LATR) employed the intrinsic infrared absorbance of tissues to facilitate a “weld” behavior in skin and other tissues.^89^ Early systematic studies with tunable lasers identified tissue‐specific optimal wavelength responses and primarily acted in the NIR II window without small molecule dyes to enhance photoconversion. For example, Gayen et al. showed ex vivo that human aorta achieved optimal tensile strength at 1460 nm, while porcine and human skin were optimally sealed at 1455 nm.^90^ To enable stronger tissue approximation, high‐concentration protein solutions acting as a biologic “solder” were developed with albumin, being highly abundant and photo‐absorbing, favored in early investigations beginning in the 1980s.^112^ To improve the healing properties of albumin‐based tissue solders, Poppas et al. incorporated 50% wt/vol albumin with either HB‐EGF, bFGF, or TGF‐β1, with the aim that growth factor would be released from the solder post‐sealing and stimulate additional healing properties in wounds.^91^ Incisional wounds were made in Yorkshire swine and sealed via LATR using an albumin solder with or without growth factor using a 1320‐nm laser aiming to achieve a temperature of 70°C at a sealing rate of 0.4 mm/s. Tensile strength was measured at several time points postsurgery and demonstrated a twofold to threefold increase in tissue strength at Day 7 for albumin solder containing TGF‐β1 (~125 kPa) versus solder alone (~60 kPa) or with the other two growth factors (~50–60 kPa).^91^ Further analysis comparing sutures, laser with solder, or laser with solder containing TGF‐β1 demonstrated substantial increases in tissue strength, hydroxyproline content, and tissue bridging. Subsequent studies from the same group demonstrated that higher concentrations of albumin (50% versus 25% wt/vol) in the solder were proportional to greater tensile strength when tested in a Yucatan swine incisional wound model, demonstrating a mechanical role for albumin in tissue approximation.^92^ Whereas early studies with LATR utilized continuous wave lasers, the advent of more advanced laser technologies, such as femtosecond lasers, facilitated novel modalities of intrinsic tissue welding. Sriramoju and Alfano reported using ultrafast femtosecond pulsed 1550‐nm lasers to seal incisional wounds in a guinea pig model.^93^ Compared to the poor epithelialization, granulation, and collagen organization of sutures, continuous wave laser illumination exhibited modest improvement.^93^ However, femtosecond laser welding resulted in complete epithelialization, appropriate granulation, and near‐normal collagen deposition.^93^ We recently developed the ability for several other biopolymers, including silk fibroin, glutaraldehyde‐crosslinked chitosan, glycerol‐treated cellulose (gCellulose), and glycerol‐treated alginate (gAlginate), to be used as tissue solders by employing a continuous wave mid‐IR laser (6500 nm, 1.76 W/cm^2^).^94^ Silk fibroin films and gCellulose (13 mg films in 1‐cm incisions) were evaluated in an incisional wound healing model in Balb/c mice compared to sutures and clinical cyanoacrylate dermal glue. Compared to sutures, mid‐IR‐activated biomaterial‐sealed wounds exhibited significantly higher maximum strain before breakage (silk 76% of intact, gCellulose 86% of intact, versus 51% and 41% of intact skin strain for sutures and skin glue, respectively) and superior tissue bridging, demonstrating the benefit of the non‐ionizing stimulation of biomaterial solders in wounds.^94^

### 5.2 Gold and silver nanoparticles

Gold and silver nanoparticles can be engineered to exhibit strong NIR I absorption, facilitating strong local heating and minimizing the detrimental effects of broader tissue heating in tissue sealing. Gobin et al. described an early use of gold nanoshells (10^10^ particles/mL) as a photothermal converter supplemented into an albumin solder, thereby allowing excitation at 808 nm with a continuous wave laser instead of longer wavelengths more likely to stimulate endogenous tissue proteins.^95^ The authors found that the gold nanoshell‐supplemented albumin solder successfully sealed rat incisional wounds, which ultimately healed appropriately, albeit with overall lower tissue strength over the course of healing (~100 kPa at Day 10 and ~1500 kPa at Day 32) compared to conventional sutures (~250 kPa at Day 10 and ~3000 kPa at Day 32).^95^ In pursuit of enhancing the observed strength of mechanical approximation with sutures, Ghosh et al. developed a laser‐activated tissue‐integrating suture (LATIS), which combines extruded collagen fibers with gold nanorods (0.5% wt).^96^ LATIS were tested in a full‐thickness incisional model in Balb/c mice with in situ activation using an 808‐nm handheld laser at a rate of 0.5 mm/s for 2 min with a power density of 2.4 W/cm^2^ maintaining the temperature at approximately 65–70°C. While LATIS without laser activation achieved similar ultimate tensile strength (~62 kPa) to commercially available polyglycolic acid sutures (~50 kPa) and silk sutures (~62 kPa), photoactivation of LATIS mediated a 160% increase in tissue strength (~100 kPa) with improved epidermal thickness, bridging, and collagen deposition with minimal increases in tissue inflammation.^96^ We also developed laser‐activated nanosealants (LANS) utilizing the same gold nanorod (1% wt) photothermal properties in combination with silk fibroin as a solder or paste‐type sealant, avoiding additional tissue trauma during suture application.^97^ When tested in a full‐thickness incisional model in Balb/c and C57BL6/J mice, gold nanorod LANS excited by an 808‐nm handheld laser (2 min at 4.8 W/cm^2^, 0.5 mm/s, and approximately 60°C) rapidly sealed the skin with significantly improved tensile strength (~35 kPa) compared to skin glue (~25 kPa) and silk sutures (~25 kPa), and significantly increased tissue resilience (~17 kPa) versus silk sutures (~10 kPa) at 2 days postinjury.^97^ Li et al. further employed gold nanorods (10.5–42 mg/g) coated with polydopamine (see next section) to develop an antibacterial hydrogel with the structural component provided by a composite of polymerized *N*‐acryloyl glycinamide (PNAGA) and bacteria‐pretreated macrophage membranes.^98^ Macrophage membrane‐containing PNAGA hydrogels exhibited greater bacterial killing upon NIR stimulation in vitro versus PNAGA hydrogels without macrophage membrane. When tested in an *S. aureus*‐infected non‐splinted full‐thickness wound model in Sprague Dawley rats, in situ activation of NIR photothermal properties of the macrophage membrane‐containing PNAGA hydrogels with an 808‐nm laser at 2 W/cm^2^ for 5 min resulted in significantly accelerated wound closure with reduced inflammatory response versus control, gauze, and hydrogels without NIR stimulation.^98^ Recent studies by Pruksawan et al. demonstrate that in addition to photothermal killing of bacteria, gold nanorods (2%–7% vol/vol, concentration unknown) can act as microheaters to cure injectable hydrogels composted of poly‐2‐hydroxyethyl methacrylate (pHEMA) and GelMA, thus combining the functionality of heat‐mediated antibacterial properties with in situ biomaterial generation.^99^

Several groups have demonstrated in situ light‐activating properties for AgNPs in dermal wounds as well. Zhang et al. reported DOPA‐modified gelatin biomineralized with AgNPs (Gel‐DA@AgNPs, 1 mg/mL) with NIR responsiveness.^100^ Formulating their Gel‐DA@AgNPs with guar gum resulted in a remoldable, injectable, and self‐healing hydrogel capable of completely covering irregularly shaped wounds. When tested in an *S. aureus*‐infected full‐thickness wound healing model in Kunming mice, Gel‐DA@Ag guar gum hydrogels irradiated with an 808‐nm laser (2 W/cm^2^) for 3 min resulted in significantly accelerated healing by Day 4 postinjury versus untreated controls, guar gum alone, Gel‐DA guar gum hydrogels without AgNPs, and complete hydrogels without NIR irradiation.^100^ Ma and colleagues similarly utilized polydopamine‐decorated AgNPs (PDA@AgNPs, 200 μM) along with catechol‐modified gelatin (Gel‐Cat) and iron to develop an injectable hydrogel for in situ combined photothermal and silver‐mediated antimicrobial protection of healing wounds.^101^ The hydrogels were tested in an *S. aureus*‐infected full‐thickness wound healing model in Kunming mice with 10 min of in situ NIR irradiation using an 808‐nm laser with 1.3 W/cm^2^ power density. Interestingly, the addition of any sort of AgNP (with or without polydopamine coating or irradiation) resulted in accelerated wound healing, demonstrating a silver‐mediated antimicrobial therapy facilitating wound closure, and the addition of both polydopamine and NIR activation resulted in further enhancement, suggesting either an enhanced bacterial clearance or the induction of a photothermal biomodulatory effect on healing response.^101^

### 5.3 Polydopamine

**FIGURE 4:**
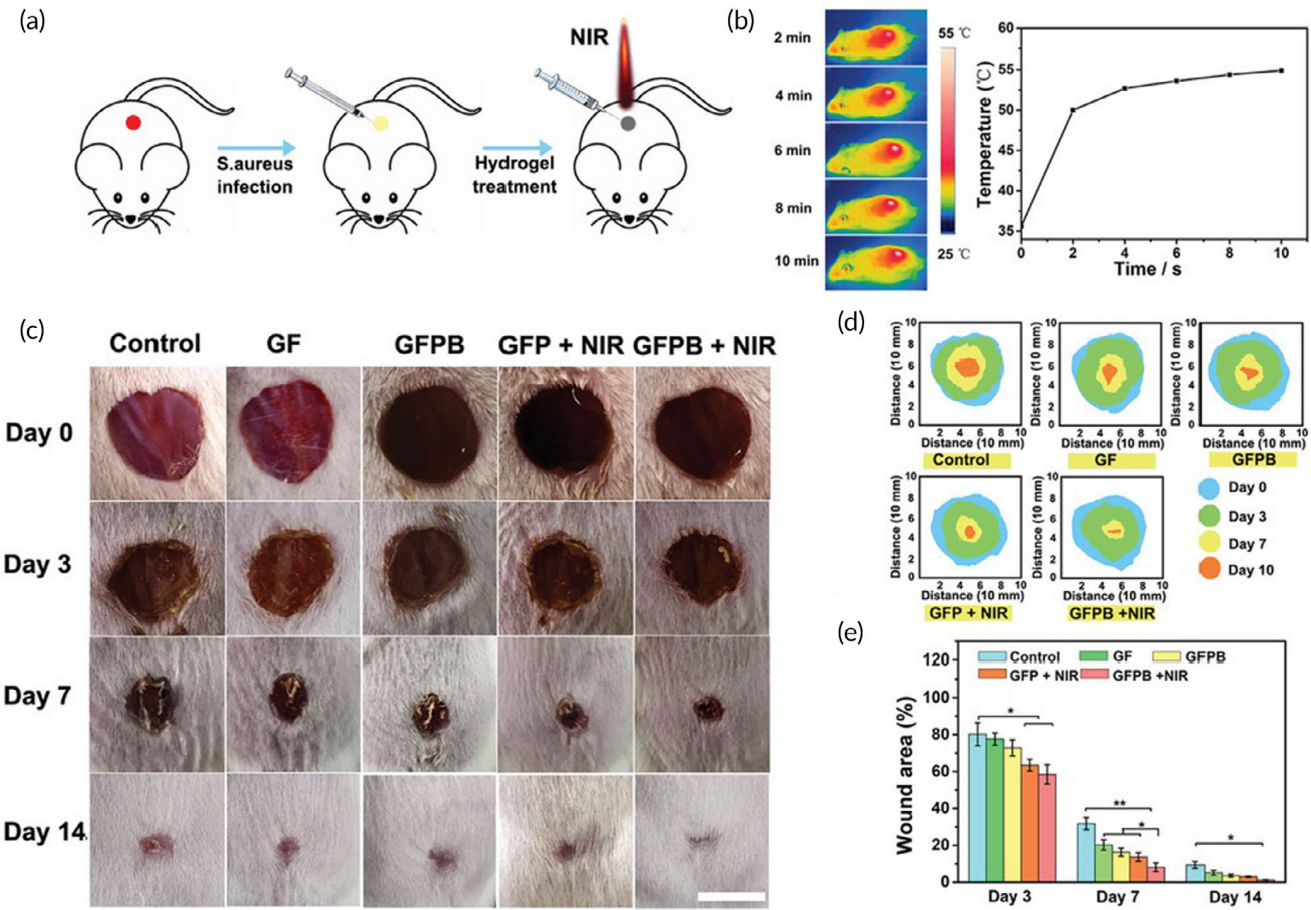
Evaluation of near‐infrared (NIR)‐activated wound dressing fabricated from two‐dimensional polydopamine nanosheets. (a) Schematic representation of the *Staphylococcus aureus*‐infected wound healing model with in situ 808‐nm NIR photostimulation at 1 W/cm^2^ for 10 min. (b) Photothermal imaging of mice treated with NIR light after hydrogel application. (c) Gross evaluation of wound healing. (d) Comparative wound closure mapping of various treatments over time. (e) Planimetric quantification of wound closure over time. Incorporation of the agent *N*,*N*′‐di‐sec‐butyl‐*N*,*N*′‐dinitroso‐1,4‐phenylenediamine (BNN6) induces an on‐demand release of nitric oxide. Reprinted from Liu et al.^103^

Polydopamine was discovered in the early 2000s while investigating the properties of ubiquitously fouling mussel organisms, which exhibited the ability to adhere to nearly all surfaces.^113^ In the course of these investigations, it was found that submersion of material in a slightly basic solution of DOPA resulted in a polymerized coating of DOPA (polydopamine) on the surface of the material, which becomes a form of NIR I‐absorbing melanin upon oxidation.^102^ Liu et al. developed two‐dimensional (2D) polydopamine nanosheets (0.2% wt/vol) loaded with the nitric oxide donor *N*,*N*′‐di‐sec‐butyl‐*N*,*N*′‐dinitroso‐1,4‐phenylenediamine (BNN6) for on‐demand release of NO upon 808‐nm irradiation from self‐assembling hydrazide‐modified gamma‐polyglutamic acid and aldehyde‐terminated Pluronic F127 hydrogels.^103^ The hydrogels were tested in an *S. aureus*‐infected full‐thickness wound model in Balb/c mice, wherein the wounds were injected with hydrogel formulations and in situ irradiated by 808‐nm laser at 1 W/cm^2^ for 10 min. While empty hydrogels exhibited a modest accelerating effect on infected wounds, NIR irradiation of the polydopamine nanosheets facilitated an enhanced effect, which was further therapeutically augmented by the addition of the BNN6 NO donor, demonstrating the therapeutic effect of both photothermal antimicrobial killing as well as NO‐mediated microbicide^103^ (Figure 4). Tao et al. similarly loaded mesoporous polydopamine nanoparticles with curcumin (0.79% wt/vol) within a dibenzaldehyde‐grafted poly(ethylene glycol) and lauric acid‐terminated chitosan hydrogel for on‐demand NIR‐mediated release of curcumin.^104^ Sprague Dawley rats were used to develop an *S. aureus*‐infected full‐thickness wound model with the hydrogel injected at the wound site and in situ activated by 808‐nm laser (1.0 W/cm^2^) for 10 min. Interestingly, almost no effect was observed for the direct administration of curcumin, the hydrogel carrier, the hydrogel with NIR irradiation, or the hydrogel containing the polydopamine nanoparticles loaded with curcumin.^104^ However, when wounds were treated with a hydrogel containing polydopamine nanoparticles containing curcumin, a nearly 200% faster rate of closure was observed on Days 3 and 7 postwounding, with complete closure achieved by Day 14 (compared with approximately 80% for all other groups).^104^ This was associated with increased collagen deposition, dramatically reduced bacterial load, and increased angiogenesis.^104^ Notably, photothermal clearance of bacteria was not observed in this study. In contrast, Guo et al. employed a hydrogel composed of polyacrylamide, polydopamine (0.16% wt/vol), and magnesium and tested its efficacy as a wound dressing in an *S. aureus*‐infected full‐thickness wound model in Sprague Dawley rats with in situ irradiation with an 808‐nm laser at 2 W/cm^2^ for 10 min.^105^ In this study, photothermal activation of polydopamine resulted in ~50% reduction in bacterial load in wounds, which was slightly enhanced by the incorporation of magnesium into the hydrogel.^105^ Similarly, wound closure and collagen deposition were enhanced with the irradiation of polydopamine in the hydrogels, which was again enhanced by the incorporation of magnesium.^105^ Extending this platform further, Wang et al. developed a multifunctional hydrogel dressing containing glucose oxidase to suppress hyperglycemia via the generation of H_2_O_2_ and gluconic acid from glucose, along with MnO_2_ nanoparticles to further catalyze H_2_O_2_ to water and oxygen within polydopamine (0.6% wt)/acrylamide hydrogels.^106^ In this system, both polydopamine and MnO_2_ nanoparticles exhibited photothermal activity in the NIR range. When tested in a streptozotocin‐induced diabetes‐impaired wound healing model in ICR mice with 10 min of in situ 808‐nm irradiation, the authors observed an additive effect of both the glucose oxidase and MnO_2_ components of the hydrogel, with the combination achieving the greatest wound closure rate by Day 14 postinjury.^106^ While the antimicrobial properties of this platform were only tested in vitro in this study, bacterial burden is a common complication of diabetic wounds and it may be hypothesized that this platform would find efficacy in infected diabetic wounds as well.^106^

### 5.4 Upconverting nanoparticles

Upconverting nanoparticles (UCNPs) are lanthanide‐ or actinide‐doped photon upconverters capable of transitioning NIR light to visible light for biomedical photonic applications.^107^ Han et al. employed UCNPs with poly(allylamine) modification in a hyaluronate‐rose Bengal complex for photochemical bonding by NIR illumination in deep tissues.^108^ The authors utilized NaYF_4_:Yb/Er UCNPs to convert 980‐nm NIR I/II laser illumination to green light in an incisional wound model in the deep tissue of Balb/c mice with in situ activation for 20 min at 500 mW/cm^2^ at NIR or green light at 7.5 mW/cm^2^. Incisions sealed with green light exhibited significantly increased tensile strength at 3 days postinjury (~17 kPa), which was more than doubled when illuminated with NIR light (~35 kPa), demonstrating the benefit of longer wavelengths to activate deep tissue‐localized photoactivated adhesives.^108^ Ma et al. modified a UCNP‐based wound dressing using cerium oxide (CeO_2_)‐modified LiYbF_4_:Tm^3+^@LiYF_4_ nanoparticles (200 μg/mL) within polycaprolactone membranes to facilitate deep tissue NIR‐to‐UV‐activated ROS generation.^109^ The authors tested their dressings in both diabetic and infected wounds. For diabetic‐impaired wounds, the authors placed their dressings in full‐thickness wounds on streptozotocin‐treated Sprague Dawley rats followed by 15 min of 980‐nm irradiation at 1.0 W/cm^2^ and observed an early acceleration of healing at Days 3 and 5 postinjury, with a modest acceleration by day 15.^109^ The mild therapeutic improvement in this model may be due to the lack of splinting on the wounds, which allowed wound contraction. In *S. aureus*‐infected wounds in Sprague Dawley rats treated in situ with 15 min of 980‐nm light at 1.0 W/cm^2^, the authors observed accelerated wound closured throughout the healing timeline as early as Day 3, with robust bacterial clearance beginning on Day 5 postinjury.^109^

### 5.5 Metal sulfide nanoparticles

Several metal sulfide nanoparticle structures have been utilized as NIR photoconverts in wound dressings. Yang et al. used tungsten disulfide nanosheets (2 mg/mL) in a chitosan hydrogel with ciprofloxacin to evaluate multifunctional stimulation of healing in infected wounds.^114^ Interestingly, the authors elected for a delayed in situ light activation of their materials, allowing infection to take hold rather than kill the bacteria immediately postinoculation. Full‐thickness wounds were generated in Balb/c mice and inoculated with *S. aureus* for 1 day, with NIR activation of placed hydrogels occurring on the subsequent day with an 808‐nm laser at 0.5 W/cm^2^ for 10 min.^114^ At 5 days postinjury, the authors observed an equivalent wound healing rate with their complete hydrogel combined with NIR I irradiation to uninfected wounds.^114^ Comparatively, an intermediate healing rate was observed with their hydrogel without irradiation, hydrogel alone, or hydrogel with nanosheets without irradiation.^114^ Yan et al. prepared a PVA hydrogel incorporating molybdenum disulfide (MoS_2_)‐polydopamine‐AgNPs (7.5 mg/mL), observing that MoS_2_ and polydopamine enhanced the strength of their PVA hydrogels.^110^ The authors investigated a mixed microbial wound infection model in Kunming mice, wherein both *S. aureus* and *Escherichia coli* were inoculated into full‐thickness wounds prior to application of their hydrogels followed by 15 min of 808‐nm in situ light activation.^110^ The authors observed gross evidence of accelerated wound closure by 6 days postinjury continuing to 8 days.^110^ Jia et al. synthesized CuS nanodots (8–32 mM) within a gelatin host matrix (Gel‐CuS) followed by crosslinking with oxidized dextran (ODex) to develop a Gel‐CuS/ODex hydrogel with adhesive and self‐healing properties.^111^ The authors investigated their hydrogels in a full‐thickness *S. aureus*‐infected wound model in Sprague Dawley rats with in situ irradiation of their hydrogel with a 1064‐nm NIR II laser at 0.77 W/cm^2^ for 3 min. Interestingly, irradiation only imparted benefit over non‐irradiated hydrogels at Day 7 postwounding, with hydrogels directly providing overall healing benefit at 10 and 14 days postinjury while hydrogels without CuS exhibited no benefit beyond Day 3 postinjury, suggesting an intrinsic, non‐photoactivable therapeutic effect of Gel‐CuS/ODex independent of photothermal effects.^111^

### 5.6 Cyanine dyes

Cyanine dyes are commonly used in the biomedical sciences for far‐red and NIR microscopy. ICG is an FDA‐approved cyanine dye with diagnostic applications in cardiac, hepatologic, gastroenterological, neurologic, and ophthalmic angiography.^115^ Kirsch et al. demonstrated a formulation of albumin‐based protein solder augmented by doping with ICG (2 mg/mL) to enhance photothermal properties.^116^ Using a rat skin flap model versus open wounds and sutures, the authors found that their ICG‐doped albumin solder facilitated significantly higher closure strength at Day 0 and 3 postinjury, with enhanced healing throughout the 21‐day follow‐up period when activated in situ with an 810‐nm laser at 31.8 W/cm^2^ with a pulse duration of 0.5 s, a pulse interval of 0.1 s, and total laser activation time of 1 min.^116^ We recently reported a silk fibroin‐based laser‐activated sealant (LASE) activated by impregnated ICG dye (0.67 mg/1 cm^2^ film) and loaded with vancomycin to limit surgical infections.^117^ When tested in vivo in a Balb/c model of full‐thickness incisions, LASE containing ICG activated in situ with an 808‐nm laser at 4.5–5.5 W/cm^2^ for 20 s exhibited significantly enhanced tensile strength (~70 kPa) versus commercial sutures (~40 kPa) at Day 3 postinjury.^117^ Further, vancomycin released from the LASE facilitated a significant 3–4‐log reduction in MRSA load in infected wounds, whereas the photothermal effect from the ICG in the LASE did not affect MRSA load.^117^ Wang et al. recently reported a gelatin and collagen hydrogel containing antimicrobial peptides conjugated to the heptamethine cyanine dye cypate (120 μM) and liposome‐encapsulated perfluorodecalin as an oxygen carrier.^118^ The authors investigate their hydrogel in Balb/c mice with full‐thickness wounds infected with *S. aureus* with in situ irradiation at 808 nm (1.5 W/cm^2^, 6 min). Interestingly, the authors observed microbial clearance with perfluorodecalin and antimicrobial peptides with or without NIR activation, but found a NIR‐dependent acceleration of wound healing only with the full hydrogel formulation, suggesting multiple paths to enhance reparative or immunologic support of healing infected wounds.^118^

### 5.7 Modified carbohydrates

Cellulose and starch describe linear and branched polymers of glucose, respectively, which can form the basis of a wide variety of biomaterials. Mao et al. developed a shape memory material based on the aldehyde groups of oxidized starch (4.8%–6.4% wt) mixed with gelatin linked in a Schiff base reaction to create shape memory net points.^119^ Their material holds a stretched state that contracts and recovers its initial size upon heating to 38°C with infrared light. The authors generated linear incisional wounds in New Zealand white rabbits and affixed their oxidized starch‐gelatin (OSG) dressings to the wounds using a dermal glue.^119^ Upon IR activation in situ to 38°C, the materials contracted and approximated the edges of the wound. Comparing to untreated wounds, sutures, or silicon wound dressings, the OSG dressings maintained the most durable wound approximation, allowing superior tissue bridging and significantly reduced wound depth by Day 7 and continuing to Day 10 postinjury.^119^ Chen et al. developed a nanocage wound dressing using cellulose nanofibers (0.24% wt) prepared via TEMPO oxidation and converted to a hydrogel with poly(vinyl alcohol).^120^ In vitro investigation confirmed that the cellulose nanofibers exhibited antimicrobial photothermal effect against *S. aureus* biofilms upon 808‐nm NIR irradiation, which was further augmented by the inclusion of ICG for in vivo study.^120^ The authors developed two full‐thickness wound healing models in Balb/c mice using *S. aureus* and drug‐resistant *S. aureus* with in situ activation of their hydrogels using 15 min of 808‐nm laser illumination. While the authors observed that *S. aureus*‐infected wounds healed more rapidly in the presence of their hydrogel with or without NIR irradiation by Days 3 and 7 postinjury, the overall effect was no different than control animals over 14 days of healing.^120^ Interestingly, drug‐resistant *S. aureus* infection resulted in a drastically delayed healing course over 14 days in control animals, which was significantly accelerated to near‐complete healing in 10 days with the use of cellulose nanofilm dressings irradiated by NIR light.^120^ In both cases, collagen volume fraction was increased at Days 3 and 14 postinjury, demonstrating a difference between the hydrogel matrix‐mediated collagen deposition activity and the antimicrobial proclosure properties of the photothermal activity of the dressing.^120^ The same group further demonstrated that decorating cellulose nanocrystals (2.6% wt) with ICG or DOPA can enhance the NIR responsiveness of CNC with significantly enhanced closure of MRSA‐infected wounds by Day 10 postinjury in a full‐thickness wound model in Balb/c mice treated in situ with 808‐nm irradiation.^121^

### 5.8 Other NIR photosensitizers

Several other photosensitizers have been utilized to convert NIR irradiation to prohealing properties in dermal wounds. Yu et al. functionalized a chitosan/silk fibroin (CS/SF) cryogel scaffold with tannic acid and ferric iron (150–1000 μg/mL) to imbue NIR photothermal responsiveness and concentration‐dependent increases in compressive strength.^122^ The authors evaluated the ability of their CS/SF‐based scaffolds to enhance acute (noninfected) wounds in a non‐splinted full‐thickness wound model in Sprague Dawley rats. The incorporation of tannic acid and ferric iron to the CS/SF scaffolds in the absence of NIR I activation imparted a prohealing effect by 6 days postwounding, underscoring the potential benefit for the compressive property augmentation by the tannic acid and iron.^122^ When the wounds were infected with *S. aureus* and treated with the cryogels with or without 808‐nm irradiation at 2 W/cm^2^ for 5 min, no effect was observed for the CS/SF cryogels alone or containing tannic acid and ferric iron, or for CS/SF cryogels irradiated with NIR, but significant acceleration of closure was observed for CS/SF cryogels with tannic acid and ferric iron combined with NIR irradiation.^122^ MXene is another, novel metal material with 2D properties and particularly high photothermal conversion efficacy. Jin et al. utilized MXene to develop a complex NIR‐responsive wound dressing by constructing a PLGA and MXene‐cored (spin‐coated, concentration not reported), DOPA‐hyaluronic acid‐shelled hydrogel with encapsulated VEGF growth factor and a diallyl trisulfide (DATS) H_2_S donor.^123^ When tested in a non‐splinted full‐thickness wound model in Balb/c mice, the MXene‐based dressing alone exerted no prohealing effects on the skin, but significant wound closure was observed with in situ irradiation at 808 nm (0.33 W/cm^2^) for 3 min, allowing the release of VEGF and DATS and stimulating a photothermal effect.^123^ Interestingly, NIR irradiation daily for 7 days was superior with respect to collagen deposition versus daily for 14 days, suggesting a detrimental effect of photothermal treatment beyond a threshold.^123^ Poly(3,4‐ethylenedioxythiophene):poly(styrene‐sulfonate) or PEDOT:PSS is an aqueous conductive polymer nanoparticle that exhibits high photothermal efficacy with NIR irradiation.^124^ Xue et al. developed a hydrogel containing quaternized chitosan and oxidized hyaluronic acid containing PEDOT:PSS (2 mg/mL) as a photothermal converter to generate an antibacterial strengthened wound dressing loaded with berberine and EGF.^125^ The hydrogels were evaluated in a non‐splinted full‐thickness wound model in Kunming mice infected with *S. aureus*, with some groups receiving in situ irradiation at 808 nm (2 W/cm^2^) for 10 min. The authors observed a drug‐ and NIR‐dependent reduction in wound size during the early stages of healing at 4 days postinjury, with the combination of berberine, EGF, and NIR superior to hydrogels exposed to NIR alone, containing berberine alone, or berberine with NIR.^125^ Recently, Wang et al. developed a series of chitosan‐based scaffolds containing carbon nanotubes (2% wt/vol) as photothermal converters and hydroxyapatite with high swelling ratio, antibacterial activity and cyto‐ and blood compatibility.^126^ The authors evaluated the hydrogel series in a non‐splinted full‐thickness wound healing model in Balb/c mice with or without in situ irradiation with 808 nm light for 20 s.^126^ They observed that while all of the hydrogels (chitosan alone, chitosan with carbon nanotubes, or chitosan with carbon nanotubes and hydroxyapatite), the combination of all three components was superior for wound closure rate as well as collagen deposition, though only the closure rate was dependent on NIR irradiation.^126^

## 6 CONCLUDING REMARKS AND FUTURE DIRECTIONS

The wide variety of in situ light‐activated dressings summarized here underscore the broad range of possibilities for novel formulations to address complex and hard‐to‐treat dermal wounds. This review focuses specifically on dressings in which proof‐of‐principle has been demonstrated in vivo in a preclinical model, but there remains a huge expanse of in vitro tested light‐activated biomaterial dressings (as with all categories of experimental wound dressing) that have yet to be evaluated in an animal model and thus lack the prospective data for wide adoption.^127^ While movement into the clinical setting is understandably a long‐term goal and not expected in the early stages of any project, it is unclear why evaluating in situ light‐activated wound dressings is so infrequently brought forward to preclinical models. These models tend to be low‐cost, technically simple, and highly amenable to testing in situ light activation because of the topical accessibility of the wound (i.e., not requiring highly complex surgical techniques). Indeed, wound healing can be modeled in animals using a wide variety of techniques beyond incisional wounds and excisional punch biopsies, including skin stripping, suction blisters, abrasions, laser ablation, dermabrasion, dermatomy, and burns, which can provide diverse opportunities to understand how novel wound dressings may perform in vivo.^128^ Even evaluation of simple wound closure rate parameters and collagen staining can provide key insights into the properties of novel materials for wound dressing development.^129^ However, because so many studies are only theoretical regarding application and don't continue beyond in vitro cytotoxicity studies, these findings remain unknown. Thus, this review provides an up‐to‐date description of only a fraction of the possible approaches to generate in situ light‐activated biomaterial wound dressings. A major opportunity, then, for researchers developing novel wound dressings is to consider even a superficial investigation of performance in vivo. The field will broadly benefit from understanding how these newly developed biomaterial formulations will behave in appropriate applications, and this can best be contributed at the time of the initial report.

Not considered in detail here are the effects of light and heat on stimulating healing via photobiomodulation, photodynamic effect, and photothermal therapy, which have been reviewed by others.^130^, ^131^, ^132^, ^133^, ^134^, ^135^ Furthermore, while we have discussed some considerations associated with wavelength selection in the design of light‐activated biomaterials, systematic investigation of factors such as heat generation is not sufficiently covered in the literature for the various photoinitiators covered here, and quantitative determination of this and other aspects of tissue compatibility (e.g., toxicity, fibrotic potential, innate, and adaptive immune response) for the various agents would represent a valuable resource.

An under‐acknowledged limitation of the wound dressing development field, as it exists currently, is the reliance on wound closure (planimetry) to determine efficacy for promoting wound healing. Wound dressings can obscure the accurate determination of wound boundaries, and it is increasingly recognized that wounds that have “closed,” even diabetic wounds, may have impaired barrier function recovery.^136^ The question remains: what is a healed wound? One approach that can be pursued in incisional wound studies is ultimate tensile strength. Some, but not all, of the studies presented in this review that pursued preclinical validation in incisional wound models, evaluated skin strength (as a measure of ultimate tensile strength given in kPa), and where available, we have presented the values inline with the study summary. However, for all excisional wound models that we have highlighted, planimetry was the sole measure of wound closure efficacy. For these wounds, which are not easily measured by ultimate tensile strength, future work would benefit from functional healing measures such as transepidermal water loss, which has commonly been used in measuring skin barriers in atopic dermatitis^137^ and psoriasis^138^ studies where healing is not apparent by gross visualization, but which is not yet widely adopted in wound healing studies.^139^

Despite the extensive work in this space and several examples of laser welding being used in other tissue clinical (e.g., laser fallopian tubule ligation), commercial dressings that are light‐activated in situ for dermal wounds remain unavailable. In contrast, periodontal wounds have been addressed clinically with commercially available in situ UV curing agents for more than 20 years.^140^ Several of the agents discussed in this review are already FDA‐approved in other forms (e.g., ICG eye drops, silk fibroin sutures, collagen wound dressings, etc.), and it may be expected that in the coming years, utilization of better preclinical models will provide improved insights into translational efficacy of experimental dressings. Major barriers to clinical translation of wound healing studies in rodents have been discussed^141^ and may partly be due to the lack of utilizing anticontraction splints to force human‐like secondary intention healing in the otherwise thin rodent skin.^142^ As described above, few studies investigating experimental in situ light‐activated biomaterials have adopted the splinted model, and thus, reported findings may be difficult to deconvolve from the wound contraction rodent skin is subject to. Encouragingly, some groups have begun to use porcine skin models, which are the superior preclinical model for evaluating human‐like healing.^143^ In addition, advancements in novel microphysiological systems to model wound healing may help to achieve animal‐free experimental opportunities that closely represent human wound dynamics.^144^ Future investigations utilizing the photosensitizers described here, as well as novel photothermal and photochemical sensitizers with new functionalized biomaterial constituents, will benefit from careful consideration of biological factors in healing wounds beyond wound contraction, including immune response phenotyping, reparative response characterization (e.g., myofibroblast activation, epithelial‐to‐mesenchymal transition in the epidermis, angiogenesis), and consideration of model development factors (e.g., use of splints, dose and timing of infection establishment, etc.).^4^, ^5^, ^6^, ^145^, ^146^

## AUTHOR CONTRIBUTIONS

**Jordan R. Yaron:** Conceptualization (lead); data curation (lead); funding acquisition (equal); supervision (equal); visualization (lead); writing – original draft (lead); writing – review and editing (equal). **Mallikarjun Gosangi:** Conceptualization (supporting); writing – original draft (supporting). **Shubham Pallod:** Conceptualization (supporting); writing – original draft (supporting). **Kaushal Rege:** Conceptualization (lead); funding acquisition (equal); supervision (lead); writing – review and editing (equal).

## CONFLICT OF INTEREST STATEMENT

Jordan R. Yaron is affiliated with Vivo Bioconsulting, LLC and Endotat Biotechnologies, LLC. Kaushal Rege is affiliated with Synergyan, LLC and Endotat Biotechnologies, LLC. Other authors declare no conflict of interest.

## Data Availability

Data sharing not applicable to this article as no datasets were generated or analyzed during the current study.
